# Rhinovirus-induced VP1-specific Antibodies are Group-specific and Associated With Severity of Respiratory Symptoms

**DOI:** 10.1016/j.ebiom.2014.11.012

**Published:** 2014-11-18

**Authors:** Katarzyna Niespodziana, Clarissa R. Cabauatan, David J. Jackson, Daniela Gallerano, Belen Trujillo-Torralbo, Ajerico del Rosario, Patrick Mallia, Rudolf Valenta, Sebastian L. Johnston

**Affiliations:** a Division of Immunopathology, Department of Pathophysiology and Allergy Research, Center for Pathophysiology, Infectiology and Immunology, Medical University of Vienna, Vienna, Austria; b Airway Disease Infection Section, National Heart & Lung Institute, Imperial College London, UK; c MRC and Asthma UK Centre in Allergic Mechanisms of Asthma, London, UK

**Keywords:** RV, Rhinovirus, COPD, Chronic obstructive pulmonary disease, ICAM-1, Intercellular adhesion molecule 1, LDL-R, Low density lipoprotein receptor, ICS, Inhaled corticosteroids, SABA, Short-acting β2 agonists, PEF, Peak expiratory flow, MALDI–TOF, Matrix-assisted laser desorption/ionization–time-of-flight mass spectrometry, ELISA, Enzyme-linked immunosorbent assay, HRP, Horseradish peroxidase, O.D, Optical density, HSA, Human serum albumin, MBP, Maltose binding protein, TCID_50_, Tissue culture 50% infective dose, Rhinovirus, Asthma, Recombinant rhinovirus coat protein, Antibody response, Serological test

## Abstract

**Background:**

Rhinoviruses (RVs) are a major cause of common colds and induce exacerbations of asthma and chronic inflammatory lung diseases.

**Methods:**

We expressed and purified recombinant RV coat proteins VP1-4, non-structural proteins as well as N-terminal fragments of VP1 from four RV strains (RV14, 16, 89, C) covering the three known RV groups (RV-A, RV-B and RV-C) and measured specific IgG-subclass-, IgA- and IgM-responses by ELISA in subjects with different severities of asthma or without asthma before and after experimental infection with RV16.

**Findings:**

Before infection subjects showed IgG_1_ > IgA > IgM > IgG_3_ cross-reactivity with N-terminal fragments from the representative VP1 proteins of the three RV groups. Antibody levels were higher in the asthmatic group as compared to the non-asthmatic subjects. Six weeks after infection with RV16, IgG_1_ antibodies showed a group-specific increase towards the N-terminal VP1 fragment, but not towards other capsid and non-structural proteins, which was highest in subjects with severe upper and lower respiratory symptoms.

**Interpretation:**

Our results demonstrate that increases of antibodies towards the VP1 N-terminus are group-specific and associated with severity of respiratory symptoms and suggest that it may be possible to develop serological tests for identifying causative RV groups.

## Highlights

• Increases of rhinovirus-specific antibodies are surrogate markers for severity of rhinovirus-induced respiratory symptoms.
• Serological tests based on recombinant rhinovirus coat protein fragments identify the culprit rhinovirus strain.
• Identification of the most relevant RV strains by serology should allow the rational design of RV vaccines.

## 1 Introduction

Rhinoviruses (RVs) are the most common cause of respiratory illnesses in children and adults of all ages and are responsible for more than 50% of acute exacerbations of asthma and chronic obstructive pulmonary disease (COPD) (Donaldson et al., 2003, Johnston et al., 1995, Nicholson et al., 1993). Severe RV infections during infancy have also been linked to the subsequent development of asthma and the presence of rhinovirus in the lower airway to the contribution to disease persistence and severity (Jackson et al., 2008, Wos et al., 2008).

Additional support for the causal relationship between RV infections and asthma exacerbations comes from studies in which asthmatic and healthy individuals are subjected to experimental RV infections, in particular using RV16 as a model strain. Using such infection studies it has been demonstrated that RV infections increase airway hyper-reactivity and late phase asthmatic responses (Lemanske et al., 1989). Furthermore, experimental RV infection increases biological and physiological markers indicative for asthma (Bardin et al., 2000, Grunberg et al., 1997, Message et al., 2008). Interestingly, it was found that high allergen-specific IgE levels increase the risk of rhinovirus-induced wheezing and that rhinovirus infections increase asthmatic reactions induced by subsequent allergen provocation providing possible links between RV- and allergen-induced asthma (Calhoun et al., 1994, Soto-Quiros et al., 2012).

Using RV89 as a model we discovered recently that IgG_1_ and IgA antibodies of RV-infected individuals recognize primarily the N-terminal portion of the rhinovirus capsid protein VP1 (Niespodziana et al., 2012). The VP1-specific antibodies were also found to cross-react between various rhinovirus strains according to sequence homology (Edlmayr et al., 2011, Iwasaki et al., 2013).

Currently, more than 100 RV serotypes have been described. They are divided into two major species, RV-A and RV-B, based on the nucleotide sequences that encode VP1–VP4 capsid proteins (Kim and Gern, 2012). A newly classified third species, human rhinovirus C (RV-C) was found to occur worldwide and seems to be more virulent than other RVs (Bochkov and Gern, 2012). The majority of RV strains attach to the cell by the intercellular adhesion molecule 1 (ICAM-1), whereas a small number of serotypes use the low density lipoprotein receptor (LDL-R) to gain entrance into human cells (Greve et al., 1989, Hofer et al., 1994). The receptor for RV-C, however, has not yet been identified (Arden et al., 2010).

In this study we expressed and purified the coat protein VP1 from RV16, 89 (RV-A), 14 (RV-B) and C (RV-C) as well as the major epitope-containing N-terminal portions of the VP1 proteins to compare the kinetics, specificities and magnitudes of antibody responses in individuals with and without asthma after experimental RV16 infection. Our study revealed that experimental RV infection induced a strain-specific boost of antibody production against the N-terminal epitope on VP1 which was associated with the severity of respiratory symptoms.

## 2 Methods

### 2.1 Expression and Purification of Recombinant RV Proteins and Fragments Thereof, Peptide Synthesis

Amino acid sequences of structural (VP1-VP4) and non-structural (2A, 2C, 3C, 3D) RV proteins from RV16, 14, 89 and C were obtained from the National Center for Biotechnology Information database (Supplemental Table 1: GenBank accession numbers). Complete genes or fragments thereof with codon usages optimized for *Escherichia coli* expression were synthesized with the addition of a DNA coding for a hexahistidine tag at the 3′ end (Genscript, Piscataway, NJ, USA) and cloned into either the *NdeI/XhoI* restriction sites of pET27b vector (Novagen, Madison, WI, USA) or the *EcoRI/HindIII* restriction sites of pMalC4X vector downstream of the *malE* gene of *E. coli* (New England Biolabs, Ipswich, MA, USA) (Supplemental Table 1). DNA sequences of the plasmid constructs were confirmed by restriction enzyme analysis of midi-prep plasmid DNA (Promega, Madison, WI, USA) and DNA sequencing (MWG Eurofins, Ebersberg., Germany).

Recombinant structural and non-structural proteins as well as MBP fusion proteins containing VP1 fragments were expressed in *E. coli* strain BL21 (DE3) (Stratagene, La Jolla, CA, USA). All proteins were purified by nickel affinity chromatography under denaturing conditions (Qiagen, Hilden, Germany) as described (Niespodziana et al., 2012). The purity of recombinant proteins was analyzed by Coomassie-stained SDS-PAGE and their identity was confirmed by immunoblotting using a monoclonal mouse anti-His-tag antibody 1:1000 diluted (Dianova, Hamburg, Germany). Bound antibodies were detected with 1:1000 diluted alkaline phosphatase-coupled rat anti-mouse IgG antibodies (BD Biosciences, Erembodegem, Belgium). Protein concentrations were determined using BCA Protein Assay Kit (Thermo Fisher Scientific, Rockford, IL, USA).

The non-structural 3B protein from strain 89 (VPg: GPYSGEPKPKSRAPERRVVTQ) was produced by solid phase peptide synthesis (CEM-Liberty instrument, Matthews, NC; Applied Biosystems, Life technologies, Carlsbad, CA) with the 9-fluorenyl-methoxy-carbonyl-method, using a PEG-PS preloaded resin (Applied Biosystems, Carlsbad, CA, USA). The peptide was purified by reversed-phase HPLC (Dionex UltiMate 3000 Pump, Sunnyvale, CA) using a Jupiter 4 μm Proteo 90 Å, LC column (Phenomenex, Torrance, CA, USA) and a 10–70% acetonitrile gradient. The masses of the recombinant proteins and of the synthetic peptide were determined by mass spectrometry (Microflex, MALDI–TOF, Bruker Daltonics, Billerica, MA).

### 2.2 Patients, Experimental RV16 Infection

As previously reported (Beale et al., 2014, Jackson et al., 2014), infections with RV16 were induced in 28 asthmatic patients (11 with mild asthma and 17 with moderate asthma (GINA., 2004)) and 11 healthy adult individuals in a study approved by the ethical committee of the Imperial College of London (09/H0712/59). Informed written consent was obtained from all subjects. Only adults but no children participated in the study. The healthy adult subjects were non-smoking, non-atopic and non-asthmatic volunteers aged 21–55 years (4 females, 7 males). Patients with mild asthma were aged 19–53 years (7 females, 4 males) taking only short-acting β2 agonists (SABA). The patients with moderate asthma, aged 20–54 years (8 females, 9 males) were on short acting beta agonists (SABA) plus inhaled corticosteroid therapy. Two out of 17 moderate asthmatics were not on ICS therapy but met criteria for moderate asthma. Total IgE levels were measured using ImmunoCAP technology (Phadia/Thermofisher, Uppsala, Sweden) (healthy: 14–19 IU/ml, median: 16 IU/ml; mild asthmatics: 102–739 IU/ml, median: 207 IU/ml; moderate asthmatics: 66–368 IU/ml, median: 132 IU/ml) (Beale et al., 2014, Dostaler et al., 2011). The baseline demographic and clinical characteristics of the volunteers are described in (Beale et al., 2014; Jackson et al., 2014). Experimental infection with RV16 was induced by using a 100 tissue culture 50% infective dose (100 TCID_50_) of RV16 on day 0 by nasal spray as described (Message et al., 2008). Blood samples were taken on day 0, 7, 14 and 42 days after infection. Respiratory symptoms were assessed by daily diary cards, from 2 weeks before baseline sampling until 6 weeks post-inoculation as described (Message et al., 2008). Total upper and lower respiratory scores were calculated by summing the corrected daily scores for the 2-week infection period. Healthy individuals and asthmatic patients were grouped according to their total upper and lower symptom scores (0–20, 21–40, > 41). Anonymized serum samples were analysed with the approval of the ethics committee of the Medical University of Vienna, Austria (EK1721/2014).

### 2.3 ELISA Experiments

ELISA plates (Nunc, Langenselbold, Germany) were coated with 2 μg/ml of each of the tested proteins (recombinant structural-, non-structural RV proteins, recombinant VP1 fragments, human serum albumin HSA, maltose-binding protein). Human sera were diluted 1:100 for IgG_1_ and 1:50 for IgA, IgM, IgG_2_, IgG_3_ and IgG_4_. Bound human antibodies were detected with monoclonal mouse anti-human IgA, IgM, IgG_1_, IgG_2_, IgG_4_ (BD Pharmingen, San Diego, CA) diluted 1:1000 and IgG_3_ (Sigma-Aldrich) diluted 1:5000 followed by detection with horseradish peroxidase (HRP)-conjugated sheep anti-mouse IgG antibodies (Amersham Bioscience, Freiburg, Germany) diluted 1:2000. The optical density (O.D.) values corresponding to the levels of antigen-specific antibodies were measured at 405 and 490 nm in an ELISA reader (Dynatech, Denkendorf, Germany). All measurements were performed as duplicates with a variation of less than 5%. Plate-to-plate normalization was done by including a control serum with established VP1-specific antibody levels on each of the plates (Stern et al., 2007). The reactivity to human serum albumin (HSA) determined for each of the patient's serum was used as a negative control. O.D. values greater than the double HSA value were considered positive (cut-off). No binding of detection antibodies to coated antigens was observed when measurements were performed without addition of patient's serum (buffer control).

### 2.4 Statistical Analysis

Differences between antibody levels were assessed by Mann–Whitney U or Wilcoxon test using SPSS software. Values of p < 0.05 were considered significant.

## 3 Results

### 3.1 Production and Characterization of a Panel of Recombinant RV Capsid Proteins, Fragments Thereof and non-Structural Proteins

**Supplemental Fig. 1:**
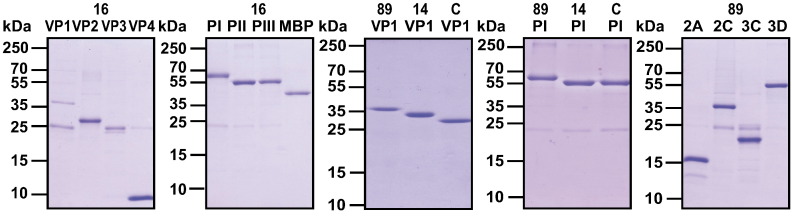
Purification of recombinant RV capsid and replication proteins. Coomassie-stained SDS-PAGE containing recombinant capsid proteins from RV16, RV89, RV14, and RVC (16: VP1–VP4; 89, 14, C: VP1), recombinant fragments thereof (16: PI, PII, PIII; 89, 14, C: PI), MBP (maltose binding protein) and non-structural proteins from RV89 (89: 2A, 2C, 3C, 3D). Molecular masses in kilo Dalton (kDa) are indicated on the left margins.

**Supplemental Fig. 2:**
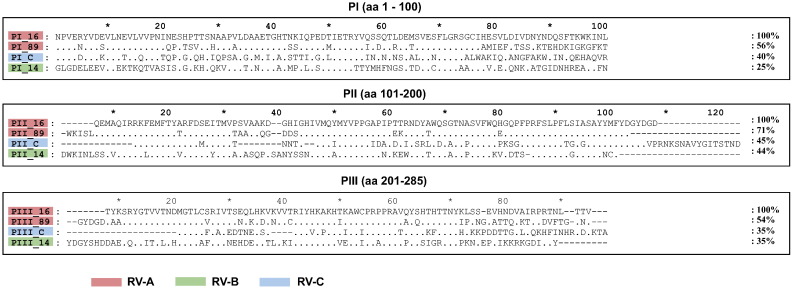
Alignment of the amino acid sequences of the VP1 fragments (PI, PII, PIII) from RV16, 89, 14 and C. Dots represent identical amino acids, dashes are gaps. Percentages of sequence identity with the RV16 sequences (top) are shown on the right margins. Red, green and blue identify RV-A, RV-B and RV-C groups, respectively.

**Fig. 1:**
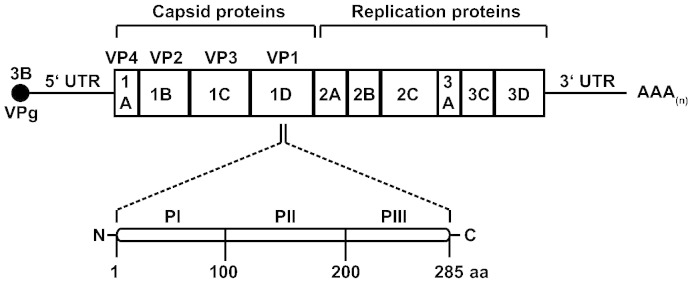
Organization of the rhinovirus genome. Genes coding for the capsid (1A, 1B, 1C, 1D) and replication proteins (2A, 2B, 2C, 3A, 3C and 3D) are indicated. The magnification shows the genes coding for three VP1 fragments (PI, PII, PIII). 3′ and 5′ UTR (untranslated regions) and the DNA coding for the peptide VPg 3B are also shown.

The scheme in Fig. 1 shows that the rhinovirus genome encodes four capsid proteins, designated VP1–VP4, and seven replication proteins (2A: protease; 2B: membrane-associated protein; 2C: NTPase; 3A: exact function is not known; 3B: protein primer for viral RNA synthesis; 3C: protease; 3D: polymerase). Supplemental Table 1 and supplemental Fig. 1 summarize the recombinant and synthetic RV proteins which were produced to monitor immune responses in patients who were inoculated with RV16. Furthermore, to investigate cross-reactivity among strains, we expressed and purified VP1 proteins and their corresponding 100 aa-fragments from RV89, RV14 and RVC (Supplemental Table 1, Supplemental Fig. 1). These proteins represent distinct rhinovirus groups, RV-A (RV16, RV89), RV-B (RV14) and RV-C (RVC). VP1 from RV89 showed a sequence identity of 63% with VP1 from RV16 whereas the sequence identity of VP1 from RVC and VP1 from RV14 to VP1 from RV16 was much lower (i.e., 44% and 38%, respectively). Likewise, PI_89 showed higher sequence identity to PI_16 (56%) than to PI_C (40%) and PI_14 (25%) (Supplemental Fig. 2).

With the exception of 2B and 3A, the remaining replication proteins of RV89 (2A, 2C, 3C, 3D) could be either expressed and purified in *E. coli* or produced as synthetic peptide (3B) (Supplemental Table 1, Supplemental Fig. 1). Each of the recombinant proteins and the 3B peptide was analysed by matrix-assisted laser desorption/ionization–time-of-flight mass spectrometry (MALDI–TOF) which confirmed their identities and calculated masses (data not shown).

### 3.2 Experimental RV16 Infection Boosts Mainly pre-Existing VP1-specific Antibody Responses

**Supplemental Fig. 3:**
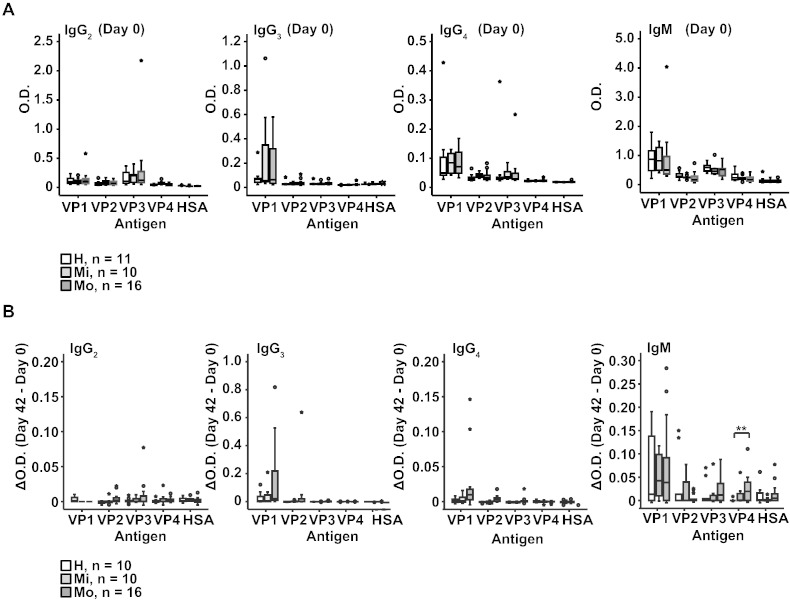
(A) IgG_2,_ IgG_3_, IgG_4_ and IgM responses to VP1, VP2, VP3, VP4, and HSA (human serum albumin) (y-axes: optical density values) are displayed as box plots for healthy subjects, mild and moderate asthmatics. Fifty % of the values are within the boxes and non-outliers are between the bars. Lines within boxes indicate median values. (B) Increases of capsid protein-specific antibody levels between day 0 and 42 are shown as in (A).

**Fig. 2:**
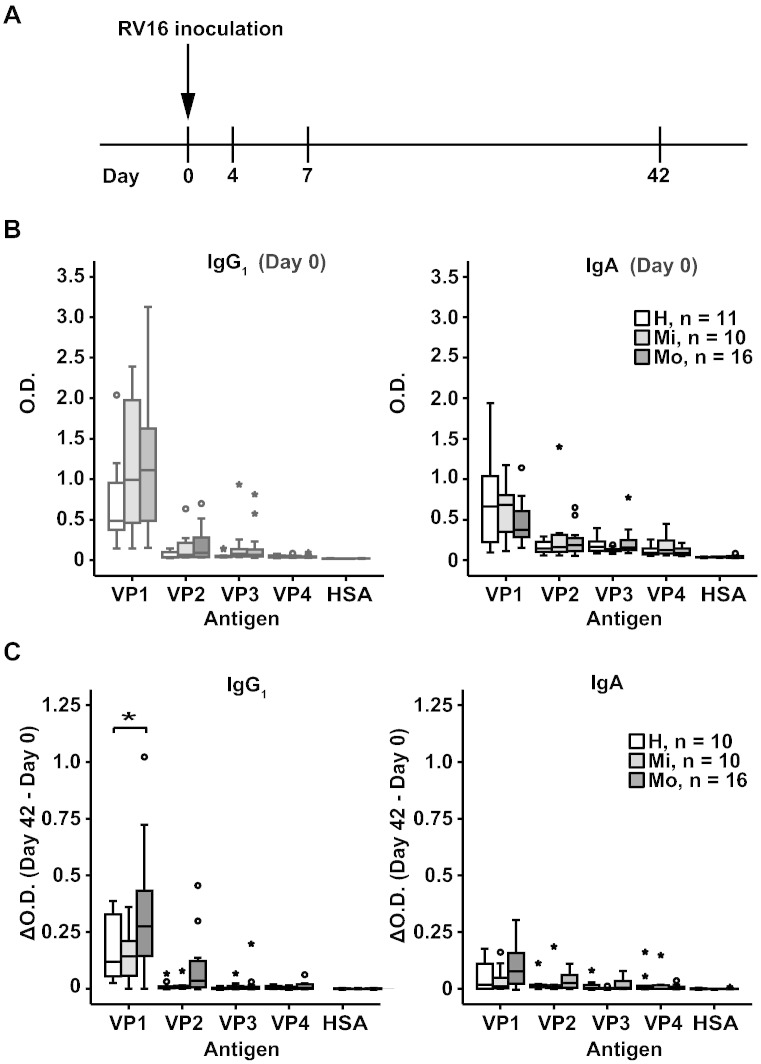
Antibody responses to recombinant RV16 capsid proteins in subjects inoculated with RV16. (A) Time course of experimental RV16 infection. Inoculation was done on day 0 and serum samples were collected on days 0, 4, 7 and 42. (B) IgG_1_ and IgA responses to VP1, VP2, VP3, VP4, and HSA (human serum albumin) (y-axes: optical density values) are displayed as box plots for healthy subjects (white bars: n = 11), mild (light grey bars: n = 10) and moderate asthmatics (dark grey bars: n = 16). Fifty % of the values are within the boxes and non-outliers are between the bars. Lines within boxes indicate median values. (C) Increases of capsid protein-specific IgG_1_ and IgA antibody levels between day 0 and 42 are shown as in (B). Significant differences are indicated (*p < 0.05).

In sera obtained on the day of virus inoculation (day 0), IgG_1_, IgA and IgM antibodies specific for VP1 were found in each of the healthy and asthmatic individuals whereas IgG_2_, IgG_3_ and IgG_4_ levels were very low (Supplemental Fig. 3). VP1-specific IgG_1_ levels were higher in the asthmatic patients than in the healthy persons but this difference was not significant. With the exception of VP2 which reacted with IgG_1_ antibodies, we found no relevant antibody responses specific for the other coat proteins (i.e., VP3, VP4) (Fig. 2B, Supplemental Fig. 3).

Next, we compared coat protein-specific antibody levels in sera obtained at days 4, 7 and 42 with those from day 0. Compared to day 0, no relevant changes of coat-protein-specific antibody reactivities were noted at days 4 and 7 (data not shown). Increases of antibody levels (ΔO.D. = O.D._Day 42_–O.D._Day 0_) compared to day 0 were only found in blood samples obtained at day 42 for VP1-specific IgG_1_ and IgA antibody levels (Fig. 2C). Interestingly, the increases of VP1-specific IgG_1_ were significantly higher in the group of moderate asthmatics compared to the healthy subjects (Fig. 2C, *p < 0.05).

**Supplemental Fig. 4:**
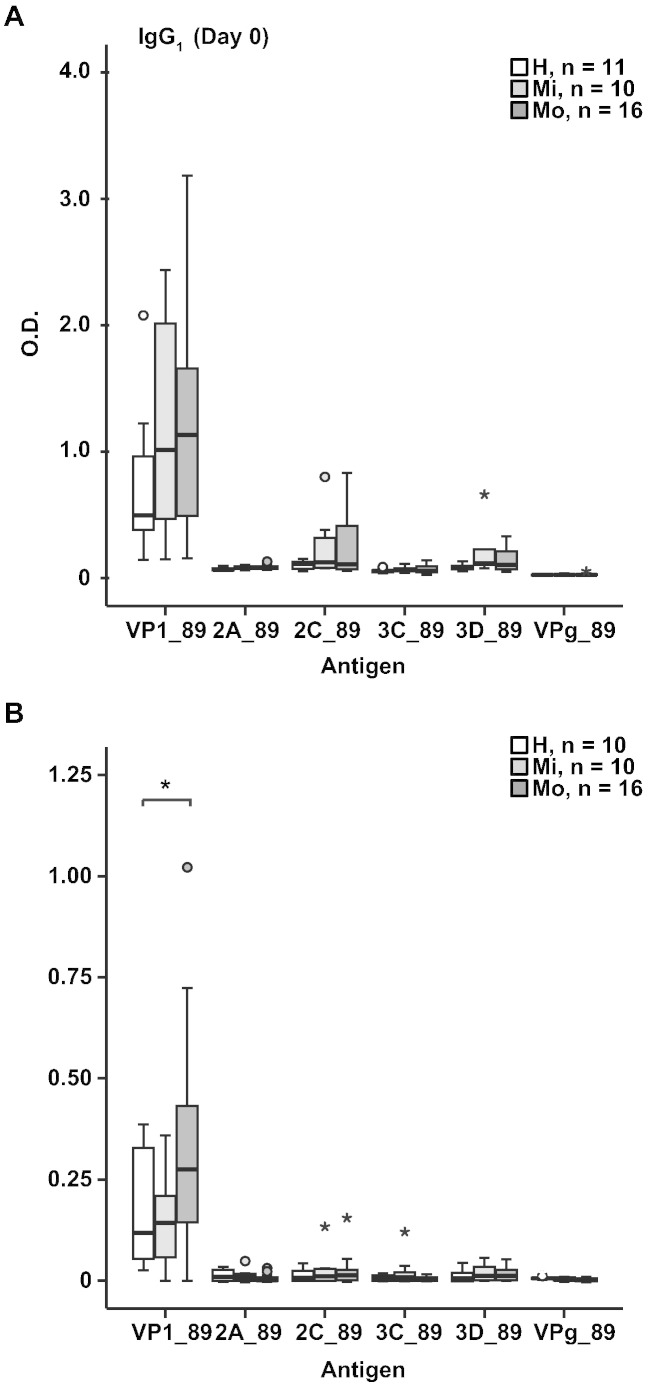
IgG_1_ antibody responses to non-structural RV proteins. (A) IgG_1_ responses to VP1 and non-structural proteins 2A, 2C, 3C, 3D and VPg from RV89 (y-axes: optical density values) are displayed as box plots for healthy subjects, mild and moderate asthmatics for day 0. Fifty % of the values are within the boxes and non-outliers are between the bars. Lines within boxes indicate median values. (B) Increases of specific IgG_1_ antibody levels between day 0 and 42 are shown as in (A).

Supplemental Fig. 4 shows that the study subjects mounted IgG_1_ responses against VP1 from RV89 and that the IgG_1_ antibodies towards VP1 from RV89 have also increased upon infection. However, we did not detect relevant IgG_1_ levels against any of the tested non-structural proteins from RV89 including 2A, 2C, 3B (VPg), 3C, or 3D and there was no relevant increase of IgG_1_ levels against these proteins upon RV infection.

### 3.3 IgG_1_ Antibodies are Mainly Directed Against the N-Terminal VP1 Fragment and Upon RV Infection Increase Strongly Among Individuals With More Severe Asthma

**Fig. 3:**
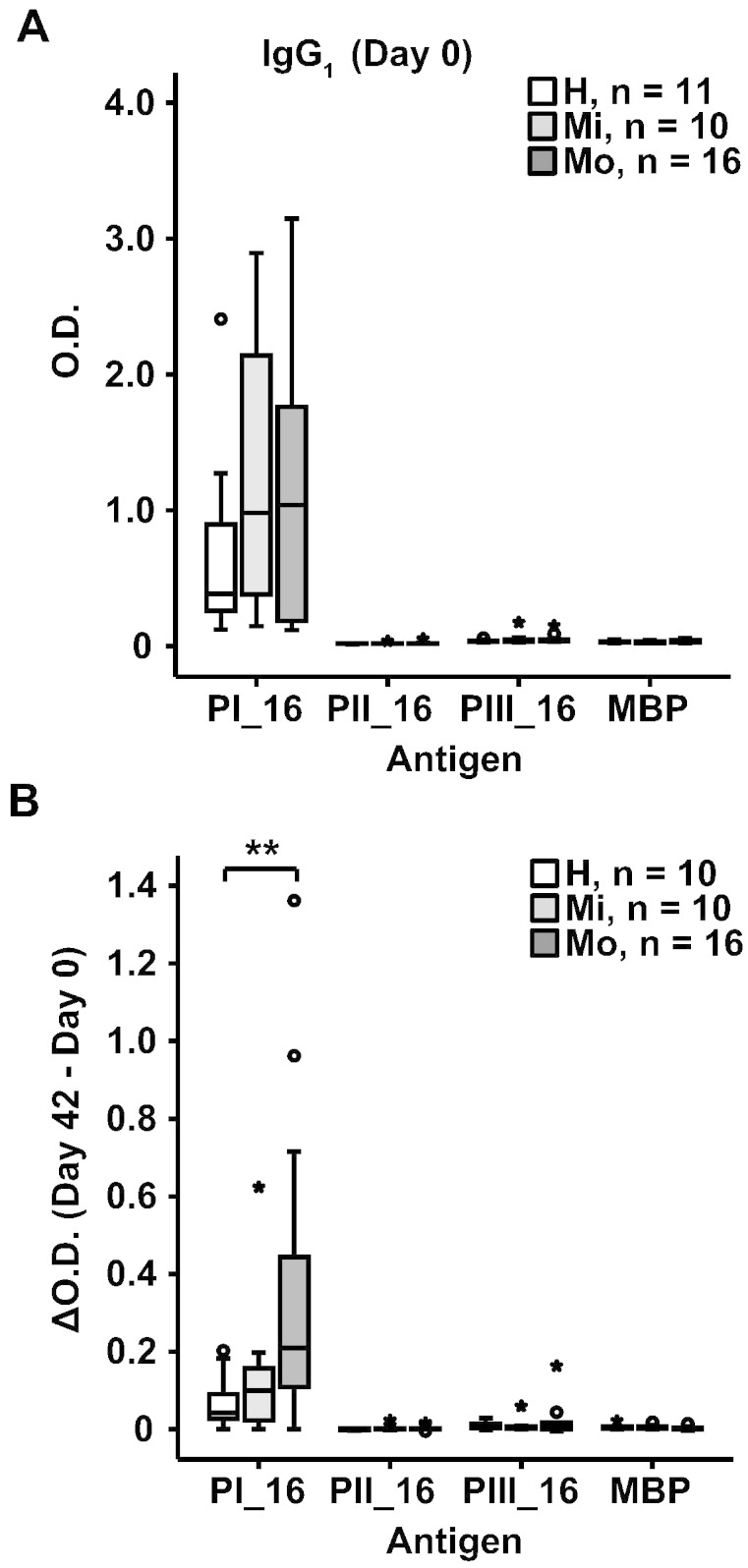
IgG_1_ antibodies are focused towards the N-terminal VP1 fragment and increase after infection. Shown are IgG_1_ levels (y-axis: optical density values) for day 0 (A) and increases between days 0 and 42 (B) for the three VP1 fragments (PI_16, PII_16, PIII_16) and maltose-binding protein (MBP) in the three groups (healthy, mild and moderate asthma). Results are displayed as box plots, where 50% of the values are within the boxes and non-outliers between the bars. Lines within boxes indicate median values. Statistically significant differences between healthy and moderate asthmatic subjects are indicated (**p < 0.01).

The epitope specificity of the VP1-specific antibodies was mapped with three recombinant fragments, of which each comprised approximately one third of RV16 VP1 (PI-PIII_16, Fig. 1, Supplemental Table 1). We found that the VP1-specific IgG_1_ responses were almost exclusively directed against the N-terminal VP1 fragment in healthy as well as asthmatic subjects (Fig. 3, upper panel). Also the increases of VP1-specific IgG_1_ antibodies were due to reactivity against the N-terminal fragment and there was no spreading of antibody reactivities towards other VP1 epitopes (i.e., PII_16 or PIII_16 fragments) (Fig. 3, lower panel). Again, rhinovirus infection boosted PI_16-specific IgG_1_ antibodies significantly stronger in the group of moderate asthmatic patients compared to healthy individuals (Fig. 3, lower panel, **p < 0.01).

### 3.4 The Increase of Antibodies After Rhinovirus Infection is Group-Specific

**Fig. 4:**
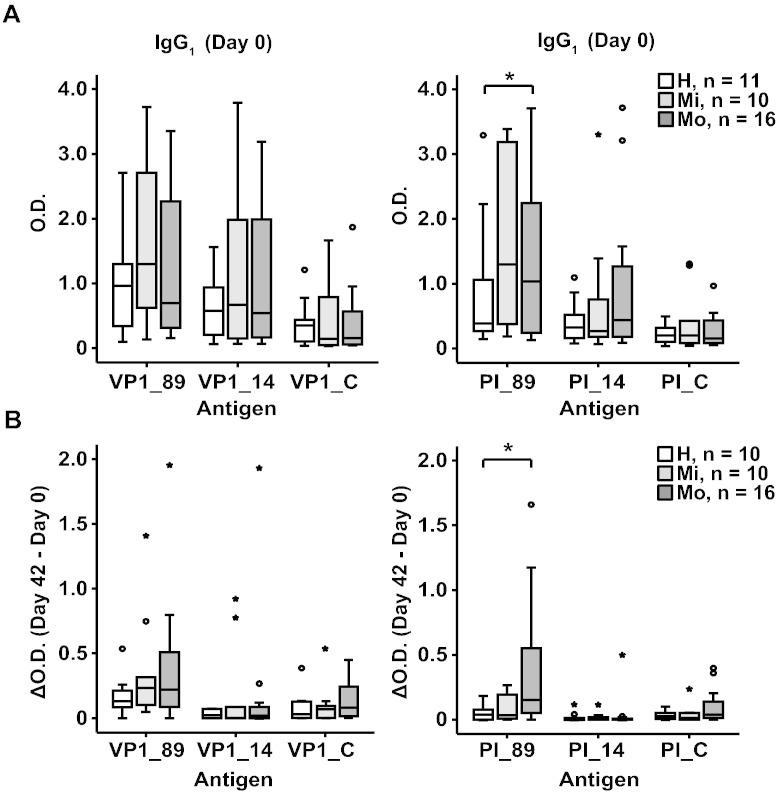
Cross-reactivity of RV16 VP1-specific antibodies. Shown are IgG_1_ levels (y-axis: optical density values) for day 0 specific for VP1 from RV89, 14 and C (left panels) and the corresponding N-terminal VP1 fragments (PI_89, PI_14, PI_C) (right panels) (A) whereas (B) demonstrates the increases between days 0 and 42. Results are displayed for the three groups (healthy, mild and moderate asthma) as box plots, where 50% of the values are within the boxes and non-outliers between the bars. Lines within boxes indicate median values. Statistically significant differences between healthy and moderate asthmatic subjects are indicated (*p < 0.05).

Fig. 4A, left panel shows that there is reactivity of IgG_1_ antibodies with VP1 proteins from RV89, RV14 and RVC in the healthy and asthmatic patients at day 0. The IgG_1_ antibodies also recognized the N-terminal fragments of the VP1 proteins from RV89, RV14 and RVC (Fig. 4A, right panel) but did not react with the PII and PIII fragments from the other strains (data not shown).

VP1 from RV14 showed the lowest degree of sequence identity with VP1 from RV16 (i.e., 38%). VP1 from RV14 and PI_14 was better recognized by the sera than the corresponding proteins from RVC at day 0 (Fig. 4A).

After infection at day 42, IgG_1_ responses increased strongest against the VP1 from RV89 which had the highest degree of sequence identity with VP1 from RV16 followed by VP1 from RVC which was the sequence next closely related whereas IgG_1_ responses to VP1 from RV14 with the lowest sequence identity showed no relevant increases (Fig. 4B). The hierarchy of increases was mirrored by the increases observed for the N-terminal fragments of the VP1 proteins although the hierarchy of the increases against VP1 and PI did not show statistical significance. However, both the PI_89 baseline IgG_1_ levels as well as their increases upon infection were significantly higher in the moderate asthmatic group compared to the healthy subjects (Fig. 4, right panels, *p < 0.05).

### 3.5 Increases of IgG_1_ Antibodies Towards the N-terminal VP1 Fragment are Highest in Patients With Severe Upper and Lower Respiratory Symptoms

**Fig. 5:**
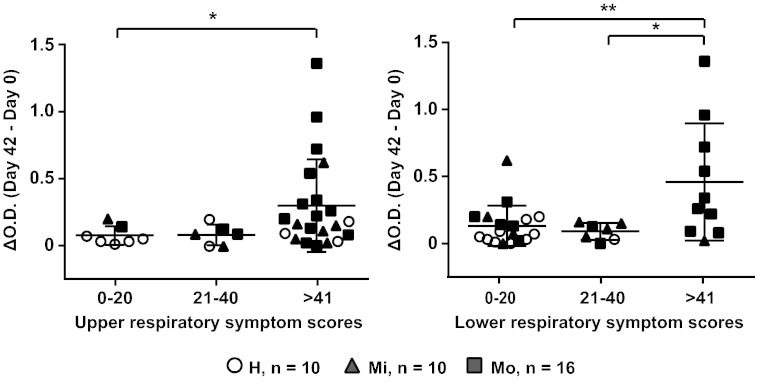
Patients with severe respiratory symptoms show higher increases of PI_16-specific antibody levels than patients with fewer symptoms. Shown are the increases (y-axes: OD levels) of PI_16-specific IgG_1_ levels in healthy controls (open circles) and patients with mild (closed triangles) and moderate (closed squares) asthma grouped according to different degrees of upper (left panel) and lower (right panel) respiratory symptoms. Significant differences are indicated (*p < 0.05; **p < 0.01).

Fig. 5 shows the associations between the increases in PI_16-specific IgG_1_ responses and subjects grouped according to upper and lower airway symptom scores recorded during infection. We found that patients with upper as well as lower respiratory symptom scores above 41 showed significantly higher increases (∆O.D. = O.D._Day 42_–O.D._Day 0_) of PI_16 IgG_1_ levels than subjects with symptoms scores up to 20 (*p < 0.05; **p < 0.01) (Fig. 5). A significantly higher increase of PI_16-specific IgG_1_ was also found for subjects with lower respiratory symptom scores above 41 as compared to subjects with a symptom score between 21 and 40 (*p < 0.05) (Fig. 5, right panel). Treatment with steroids (i.e., moderate asthmatics) seemed to have influence on the severity of lower respiratory symptoms and antibody increases in only few cases (Fig. 5).

## 4 Discussion

Here we investigated the antigen- and epitope-specificity, magnitude, course and type of antibody responses following human experimental RV16 infection. In order to study the antigen and epitope-specificity of antibody responses we expressed and purified a panel of recombinant capsid proteins and fragments thereof from four distantly related RV strains which represented the three RV groups A, B and C as well as non-structural proteins/peptides from group A. Blood samples obtained on the day of RV inoculation (i.e., baseline) showed that the antibody responses of the analysed individuals were mainly directed against a N-terminal fragment of the VP1 protein, whereas the other coat proteins VP2-VP4 as well as the non-structural proteins showed no relevant reactivity. The result obtained at baseline confirmed our earlier finding that the N-terminus of VP1 represents a major epitope of rhinovirus-specific antibodies (Niespodziana et al., 2012). Furthermore, the antibodies reacting with the VP1 N-terminal epitope belonged mainly to the IgG_1_ subclass and to the IgA class and showed cross-reactivity with the N-terminal portions of VP1 from three distantly related rhinovirus strains belonging to groups A, B and C. Among the individuals studied, the highest antibody levels were directed to group A, represented by RV 16 and 89 which may be due to the fact that these individuals were exposed mainly to rhinovirus group A strains in the past. After the experimental infection with a group A strain, RV16, we found that mainly group A-specific antibody responses increased whereas antibody responses to VP1 from groups B and C showed no (i.e., group B represented by RV14) or low (group C) increases. A correlation between antibody levels measured before and after inoculation (i.e., day 42) was observed (data not shown). It thus seemed that a boosting of an already established immune response had occurred which also lead to appearance of neutralizing activities in the sera (Jackson et al., 2014). The increases of antibody responses thus seemed to follow the degree of sequence homology among the VP1 proteins (i.e., group A > C > B) indicating that it may be possible to create diagnostic tests which may allow to discriminate between infections with different rhinovirus groups/strains based on antibody reactivity profiles towards VP1 epitopes from different rhinovirus strains. Furthermore, the analysis of blood samples at different time points after experimental inoculation showed that the increases of VP1-specific antibodies were only detectable at day 42 after inoculation but not at days 4 and 7 after infection indicating that a period of 6–8 weeks and perhaps more is required for the detection of increases of rhinovirus-induced antibody responses after infection. However, a detailed analysis of the time course of the generation of RV-induced antibody response is still needed to determine the optimal time window for antibody measurements. Another interesting observation was that increases of IgG_1_ antibody responses towards the VP1 N-terminal epitope were strongest in the group of subjects suffering from more severe forms of asthma as compared to individuals with mild asthma or without asthma. In this context, it is important to note that asthmatic individuals were observed to be more susceptible to naturally occurring RV infection than non-asthmatic subjects in that lower respiratory symptoms and changes in peak expiratory flow (PEF) were more severe and of longer duration (Bardin et al., 2000, Corne et al., 2002, Message et al., 2008, Zhu et al., 2014). When individuals were grouped according to the severity of upper and lower respiratory symptoms occurring after infection it turned out that the increases of antibody responses towards the N-terminus of VP1 were significantly higher in the individuals with severe upper and lower respiratory symptoms indicating that the antibody increases are related to the severity of respiratory symptoms. The finding that individuals with more severe manifestations of respiratory symptoms showed the highest increases of VP1-specific antibodies may be explained by their different susceptibility towards rhinovirus infections and/or by a more severe infection with greater tissue damage in the respiratory tract (Contoli et al., 2006, Wark et al., 2005). Since the N-terminus of VP1 is reported to not contain receptor binding sites for RV, these antibodies may be directed mainly against a non-neutralizing epitope and therefore may not protect against rhinovirus infections. This may explain that the individuals with more severe asthma despite mounting high VP1-specific antibody levels at baseline were not protected.

In summary our results indicate that it may be possible to use the N-terminal portions of VP1 from different rhinovirus strains to develop serological tests for the identification of rhinovirus groups/strains involved in infection. Furthermore, the increases of antibody levels may serve as surrogate markers for the severity of respiratory symptoms. At present, assays for the detection of rhinovirus strains are based on reverse transcription of viral RNA and DNA amplification by PCR. Such tests at best demonstrate the presence of certain rhinovirus strains in the respiratory tract but cannot confirm whether an infection with a certain strain has indeed taken place because it does not show whether the immune system has responded. For example, it would be important to obtain data demonstrating the role of RV-C in inducing asthma attacks as demonstrated here for RV 16, a group A strain for vaccine development. The fact that increases of VP1-specific antibodies appear late and hence are not suitable for the diagnosis of an acute RV infection may be considered as a possible limitation but on the other hand there are no RV-specific acute therapies available. However, the advantages of serological tests for identifying the culprit rhinovirus strains are that they are easy to perform and that they can be used for the retrospective analysis of serum samples collected in various asthma cohorts (Bousquet et al., 2013). Ultimately serological tests may be helpful for identifying the most common and clinically relevant rhinovirus strains involved in asthma exacerbations and to investigate in cross-sectional studies the possible role of rhinovirus infections in other respiratory diseases, in different geographic populations and age groups including not only children but also adults and elderly persons. Longitudinal studies will allow to study possible variations of culprit rhinovirus strains and to study their importance as potential vaccine components. In fact, a very recent publication showed that recombinant RV-A, -B, and -C capsid proteins were useful for the measurement strain-specific antibodies in asthmatic children (Iwasaki et al., 2014). If serological tests indeed allow identifying the most common and clinically relevant rhinovirus strains involved in respiratory diseases, this information may help designing vaccines for rhinovirus infections so that the vaccines include the clinically most relevant strains. Once such vaccines become available the tests will be useful to identify persons at risk of experiencing rhinovirus-induced exacerbations of respiratory diseases for vaccination.

The following are the supplementary data related to this article.

Supplemental Table 1 Characterization of recombinant and synthetic structural and non-structural RV proteins and fragments thereof.

## Author Contributions

KN implemented the study, analysed data, and wrote the report. RV designed the study, interpreted data and wrote the report. CC and DG made technical contributions. DJJ, BT-T, AdR, PM and SLJ recruited patients and obtained consent, and carried out the human experimental infection study. RV and SLJ were principal investigators and conceived the project. All authors read and commented on the report.

## Conflicts of Interests

RV has received research grants from Biomay AG, Vienna, Austria and serves as a consultant for this company. PM has received travel grants and speakers fees from GlaxoSmithKline. SLJ reports grants and personal fees from Centocor, grants and personal fees from Sanofi Pasteur, grants and personal fees from GSK, grants and personal fees from Chiesi, grants and personal fees from Boehringer Ingelheim, personal fees from Grünenthal, grants and personal fees from Novartis, grants, personal fees and shareholding from Synairgen, outside the submitted work. In addition, SLJ, KN and RV are authors of patent/patent applications related to the subject of the study. KN, DJ, CC, DG, AdR and BT-T declare no competing interests.
