# Supplementary material for: Rhinovirus-induced VP1-specific Antibodies are Group-specific and Associated With Severity of Respiratory Symptoms
Source: eBioMedicine. 2014 Nov 18;2(1):64–70. doi: 10.1016/j.ebiom.2014.11.012 (PMC4484518; doi:10.1016/j.ebiom.2014.11.012)
Supplement: Supplemental Table 1 — Characterization of recombinant and synthetic structural and non-structural RV proteins and fragments thereof. [file mmc1.docx]

**Supplementary Materials**

**Rhinovirus-induced VP1-specific antibodies are group-specific and associated with severity of respiratory symptoms**

**^1^Katarzyna Niespodziana, PhD, ^1^Clarissa R. Cabauatan, PhD, ^2,3^David J. Jackson, MD PhD, ^1^Daniela Gallerano, MSc, ^2,3^Belen Trujillo-Torralbo, ^2,3^Ajerico del Rosario, ^2,3^Patrick Mallia, MD PhD, ^1^Rudolf Valenta, MD and ^2,3^Sebastian L. Johnston, MD PhD**

^1^Division of Immunopathology, Department of Pathophysiology and Allergy Research, Center for Pathophysiology, Infectiology and Immunology, Medical University of Vienna, Vienna, Austria

^2^Airway Disease Infection Section, National Heart & Lung Institute, Imperial College London, ^3^MRC and Asthma UK Centre in Allergic Mechanisms of Asthma, London, UK

**Corresponding author:**

Rudolf Valenta, MD

Christian Doppler Laboratory for Allergy Research

Division of Immunopathology, Department of Pathophysiology and Allergy Research, Center for Pathophysiology, Infectiology and Immunology, Medical University of Vienna

Waehringer Guertel 18-20, 1090 Vienna, Austria

Phone: +43-1-40400-51080

Fax: +43-1-40400-51300

email: rudolf.valenta@meduniwien.ac.at

**Supplemental Table 1.** Characterization of recombinant and synthetic structural and non-structural RV proteins and fragments thereof.

| Species | Strain | Protein name | Expression vector | Lenght [aa]^a^ | Molecular weight [Da]^b^ | | GenBank accession number |
| --- | --- | --- | --- | --- | --- | --- | --- |
|  |  |  |  |  |  |  |  |
|  |  | *Capsid proteins* | | | | | |
| RV-A | 16 | VP1_16 | pET27b | 292 | 33431·5 | | AAQ19871.1 |
|  |  | VP2_16 | pET27b | 268 | 29,918·7 | | Q82122.4 |
|  |  | VP3_16 | pET27b | 245 | 26,294·2 | | Q82122.4 |
|  |  | VP4_16 | pET27b | 75 | 8,321·1 | | Q82122.4 |
|  |  | PI_16 | pMALC4X | 392 (MBP) + 107 | 43,123 (MBP) + 12,172·3 | | AAQ19871.1 |
|  |  | PII_16 | pMALC4X | 392 (MBP) + 107 | 43,123 (MBP) + 12,470·0 | | AAQ19871.1 |
|  |  | PIII_16 | pMALC4X | 392 (MBP) + 92 | 43,123 (MBP) + 10,733·2 | | AAQ19871.1 |
| RV-A | 89 | VP1_89 | pET27b | 299 | 33,599·5 | | AAQ19944.1^c^ |
|  |  | PI_89 | pMALC4X | 392 (MBP) + 107 | 43,123 (MBP) + 11,824·9 | | AAQ19944.1^c^ |
|  |  | PII_89 | pMALC4X | 392 (MBP) + 107 | 43,123 (MBP) + 12,590·1 | | AAQ19944.1^c^ |
|  |  | PIII_89 | pMALC4X | 392 (MBP) + 99 | 43,123 (MBP) + 11,128·5 | | AAQ19944.1^c^ |
| RV-B | 14 | VP1_14 | pET27b | 296 | 33,472·6 | | AAQ19869.1 |
|  |  | PI_14 | pMALC4X | 392 (MBP) + 107 | 43,123 (MBP) + 11,825·1 | | AAQ19869.1 |
|  |  | PII_14 | pMALC4X | 392 (MBP) + 107 | 43,123 (MBP) + 12,255·7 | | AAQ19869.1 |
|  |  | PIII_14 | pMALC4X | 392 (MBP) + 96 | 43,123 (MBP) + 11,335·8 | | AAQ19869.1 |
| RV-C | YP | VP1_C | pET27b | 281 | 31,834·9 | | YP001552435.1 |
|  |  | PI_C | pMALC4X | 392 (MBP) + 107 | 43,123 (MBP) + 11,907·2 | | YP001552435.1 |
|  |  | PII_C | pMALC4X | 392 (MBP) + 107 | 43,123 (MBP) + 12,229·7 | | YP001552435.1 |
|  |  | PIII_C | pMALC4X | 392 (MBP) + 81 | 43,123 (MBP) + 9,642·0 | | YP001552435.1 |
|  |  | *Replication proteins* | | | | | |
| RV-A | 89 | 2A_89 *(Protease)* | pET27b | 143 | | 16487·4 | AAA45762.1 |
|  |  | 2C_89 *(NTPase)* | pET27b | 328 | | 37079·1 | AAA45762.1 |
|  |  | 3C_89 *(Protease*) | pET27b | 190 | | 20968·8 | AAA45762.1 |
|  |  | 3D_89 *(Polymerase)* | pET27b | 467 | | 52797·6 | AAA45762.1 |
|  |  | 3B_89 *(VPg)* | synthetic  peptide | 21 | | 2339·6 | AAA45762.1 |

Abreviations: aa: amino acid; MBP: maltose-binding protein.

^a^Numbers of amino acids [aa] correspond to the protein sequences including methionine (+1) and His-tag (+6).

^b^Molecular weights were calculated according to the aa sequences using Protparam tool on the Expasy Bioinformatics Resource Portal.

^c^GenBank accession number for RV89 is given for the most similar isolate (difference of 3 aa).
